# Comparison of Single-Level Open and Minimally Invasive Transforaminal Lumbar Interbody Fusions Presenting a Learning Curve

**DOI:** 10.1155/2020/3798537

**Published:** 2020-01-22

**Authors:** Viktor Zs. Kovari, Akos Kuti, Krisztina Konya, Istvan Szel, Anna K. Szekely, Krisztian Szalay

**Affiliations:** ^1^Szent Gyorgy University Teaching Hospital of Fejer County, 3 Seregelyesi Road, 8000 Szekesfehervar, Hungary; ^2^Semmelweis University, Faculty of Medicine, 26 Ulloi Ut, 1085 Budapest, Hungary; ^3^Semmelweis University, Faculty of Medicine, Department of Orthopaedics, 26 Ulloi Ut, 1085 Budapest, Hungary

## Abstract

**Background:**

Comparison of single-level open and minimally invasive transforaminal lumbar interbody fusions (O-TLIF and MI-TLIF) of a single surgeon and presentation of his MI-TLIF learning curve in a retrospective observational cohort study.

**Methods:**

27 MI-TLIF and 31 O-TLIF patients, performed between 03/01/2013 and 03/31/2018, were compared regarding the operative time, blood loss, blood transfusion frequency, postoperative length of stay (LOS), and adverse events. An overall comparison of pre- and postoperative Oswestry Disability Index (ODI) results and Visual Analog Score (VAS) results of low back and leg pain was performed in the case of the two techniques. For a learning curve presentation, the MI-TLIF cases were compared and the optimal operative time was determined.

**Results:**

The gender ratio and age did not differ in the groups. Operative time showed no difference (*P*=0.88) between the MI-TLIF (161.2 ± 33.7 minutes) and O-TLIF groups (160 ± 33.6 minutes). Intraoperative blood loss was less (*P*=0.88) between the MI-TLIF (161.2 ± 33.7 minutes) and O-TLIF groups (160 ± 33.6 minutes). Intraoperative blood loss was less (*P*=0.88) between the MI-TLIF (161.2 ± 33.7 minutes) and O-TLIF groups (160 ± 33.6 minutes). Intraoperative blood loss was less (*P*=0.88) between the MI-TLIF (161.2 ± 33.7 minutes) and O-TLIF groups (160 ± 33.6 minutes). Intraoperative blood loss was less (*P*=0.88) between the MI-TLIF (161.2 ± 33.7 minutes) and O-TLIF groups (160 ± 33.6 minutes). Intraoperative blood loss was less (*P*=0.88) between the MI-TLIF (161.2 ± 33.7 minutes) and O-TLIF groups (160 ± 33.6 minutes). Intraoperative blood loss was less (*P*=0.88) between the MI-TLIF (161.2 ± 33.7 minutes) and O-TLIF groups (160 ± 33.6 minutes). Intraoperative blood loss was less (*P*=0.88) between the MI-TLIF (161.2 ± 33.7 minutes) and O-TLIF groups (160 ± 33.6 minutes). Intraoperative blood loss was less (*P*=0.88) between the MI-TLIF (161.2 ± 33.7 minutes) and O-TLIF groups (160 ± 33.6 minutes). Intraoperative blood loss was less (*P*=0.88) between the MI-TLIF (161.2 ± 33.7 minutes) and O-TLIF groups (160 ± 33.6 minutes). Intraoperative blood loss was less (*P*=0.88) between the MI-TLIF (161.2 ± 33.7 minutes) and O-TLIF groups (160 ± 33.6 minutes). Intraoperative blood loss was less (*p*=0.88) between the MI-TLIF (161.2 ± 33.7 minutes) and O-TLIF groups (160 ± 33.6 minutes). Intraoperative blood loss was less (*P*=0.88) between the MI-TLIF (161.2 ± 33.7 minutes) and O-TLIF groups (160 ± 33.6 minutes). Intraoperative blood loss was less (*P*=0.88) between the MI-TLIF (161.2 ± 33.7 minutes) and O-TLIF groups (160 ± 33.6 minutes). Intraoperative blood loss was less (*P*=0.88) between the MI-TLIF (161.2 ± 33.7 minutes) and O-TLIF groups (160 ± 33.6 minutes). Intraoperative blood loss was less (*P*=0.88) between the MI-TLIF (161.2 ± 33.7 minutes) and O-TLIF groups (160 ± 33.6 minutes). Intraoperative blood loss was less (

**Conclusions:**

Similar operative time and postoperative quality of life improvement can be achieved by MI-TLIF procedure as with O-TLIF, and additionally LOS and blood loss can be reduced. When comparing parameters, MI-TLIF can be an alternative option for O-TLIF with a similar complication profile. The learning curve of MI-TLIF can be steep, although it depends on the circumstances.

## 1. Introduction

Since the introduction of open transforaminal lumbar interbody fusion (O-TLIF) in 1998 by Harms and Jeszenszky, it became an internationally well-known and effectively applied procedure to achieve segmental stability with or without the decompression of the nerve elements in various lumbar degenerative pathologies [1]. The impact of extensive muscle dissection and retraction required in O-TLIF transformed the open technique into a minimally invasive (MI) one [2], introduced in 2003 by Foley et al. The MI-TLIF has the potential benefit of decreasing intraoperative blood loss, surgical site infection (SSI) incidence, time to ambulation, and postoperative length of stay (LOS) [3]. It enhances the speed of recovery with the performance of smaller incisions and therefore less soft tissue damage, resulting in clinical outcomes similar to the open procedure [3]. The introduction of a learning curve is important because MI spinal procedures, including the TLIF technique, become more and more popular, and various studies present it differently [2, 4–11].

A retrospective, nonrandomized study was conducted to evaluate the possible differences and/or similarities between single-level O-TLIF and MI-TLIF, while MI-TLIF cases were compared to display a learning curve.

## 2. Methods

From 03/01/2013 until 03/31/2018, the 58 patients who were included in our study underwent single-level consecutive MI-TLIF or O-TLIF procedures, due to the lack of response to conservative treatment. The surgeries were performed by the same surgeon (VZSK) in our institution.

### 2.1. Inclusion Criteria

- Adult patients (age > 18 years old)
- Single segmental, MI, or open TLIF procedure
- Bilateral transpedicular fixation and unilateral cage insertion
- Degenerative pathology
- Performed by the same surgeon (VZSK)

There were 13 spondylolisthesis cases (Meyerding grade I or II), 9 degenerative disc diseases (DDD) with or without spinal canal or foraminal stenosis, and nine failed back-lumbar discectomy cases involved in the O-TLIF group. In the MI-TLIF group, there were 13, 13, and one similar cases from the same type of diseases.

We conducted a study with two arms, comparing the first MI-TLIF and O-TLIF procedures to discern the differences of operative time (meaning skin to skin), intraoperative blood loss, blood transfusion frequencies, LOS, and adverse events (SSI, incidental durotomy, new motoric, sensory deficit, or cauda equina syndrome). The two techniques were compared from the point of their potential to improve the quality of life and relieve the pain as well. Numerous patients did not show up regularly for the follow-up which made it impossible to evaluate the changes in the quality of life and pain year by year properly. Therefore, we could not demonstrate accurate one-year, two-year, etc., follow-up. We decided to perform an overall comparison of Oswestry Disability Index (ODI) and Visual Analog Score (VAS) low back and VAS leg pain scores for the entire examined period. From the ODI and the VAS comparison, the four patients who acquired additional diseases (i.e., myocardial infarction, lung carcinoma, or required additional lower extremity surgery which severely influenced and worsened their physical status or increased pain level) and the three who required additional surgery due to an adjacent segment disease and received fusion extension were excluded. Thirteen patients were unreachable for follow-up. Finally, 17 O-TLIF and 21 MI-TLIF cases were used in the ODI and VAS comparisons. In each nonexcluded case, the patient's preoperative ODI and VAS scores were compared to their own postoperative scores regardless to how many years passed from the procedure until the present. The last procedure in this study was performed more than one and a half year ago, meaning that the shortest follow-up period is more than one year. First, the comparisons were made for the two groups separately; then, the two groups were compared to each other. The up-to-date ODI and VAS evaluation was conducted by interviewing the patients via phone calls.

Blood loss is only estimated because while the blood captured by a suction canister could be measured, the amount of blood wiped by sponges could not. We also compared MI-TLIF cases in terms of LOS, blood loss, and operative time year by year and determined a break point from which it became relatively constant, presenting the end of the learning curve. All the above-mentioned parameters were compared before and after the break point in the MI group.

Sextant, Longitude II (Medtronic Sofamor Danek, Memphis, TN, USA), Viper 2 (DePuy Synthes Spine, Raynham, MA, USA) in the MI and Legacy (Medtronic), and Expedium (DePuy Synthes Spine) pedicle screw-rod systems were used in addition to Capstone (Medtronic), Leopard, and T-PAL (DePuy Synthes Spine) interbody cages. In MI-TLIF, cages were inserted through an opening created by Quadrant (Medtronic) tubular expandable retractor system. Screws and rods were used always bilaterally.

The patient enrollment to either group was not randomized. Eventually, MI-TLIF procedures were preferred and promoted to the patients more often. However, it should be noted that in every case the choice between the two procedure types was based on the patient receiving full information on both types of procedures and was a shared decision between the patient and surgeon.

Most of the data was collected retrospectively from the hospital database, while the late data for ODI and VAS evaluation was collected by phone interviews.

Statistical analysis was performed using SPSS version 23.0.0 software including descriptive statistics and comparative analysis using analysis of variance (ANOVA), a test of homogeneity of variances, a Welch-d probe, Mann–Whitney *U* test, *t*-test, and a Tukey's honestly significant difference (Tukey's HSD) test. In the case of MI-TLIF procedures, we applied Rigby's and Stasinopoulos' statistical modeling [12] to determine the break point regarding the length of surgeries. Significance was defined as *P* < 0.05.

The study was approved by the Institutional Review Board.

## 3. Results

27 MI-TLIF and 31 O-TLIF consecutively treated patients were included. There was no statistical difference between the age (*P*=0.93) and gender ratio (*P*=0.07). LOS was shorter in the MI-TLIF group by 2.28 days in average than in the O-TLIF cohort (2.7 ± 1.1 vs. 5 ± 2.7 days, *P* ≤ 0.001). The length of surgery was similar in the MI-TLIF and O-TLIF groups (161.2 ± 33.7 vs. 160 ± 33.6 minutes, *P*=0.88) regarding the whole examined period. The amount of intraoperative blood loss was twofold less in the MI-TLIF group than in the O-TLIF group (288.9 ± 339.8 vs. 682.3 ± 465.4 mL, *P* ≤ 0.001) (Table 1). It must be noted that in some (5 MI and 2 open cases) procedures where the blood loss was so insignificant the anesthesiologist did not register the amount of blood loss; we substituted the value with the minimal registered volume which was 100 mL. There was no difference in blood transfusion incidence comparing the two groups (*P*=0.64). Blood transfusions were applied twice in the open group (6.45%) and once (3.7%) in the MI group (Table 1).

### 3.1. Adverse Events (Table 2)

There was no SSI in the MI-TLIF group while there were two (6.45%) in the O-TLIF group, but this result gave no significant difference (*P*=0.17). There were four (12.9%) incidental durotomies in the O-TLIF group and two (7.4%) in the MI-TLIF group, which gave no significant difference (*P*=0.5). All were repaired intraoperatively with no harmful outcome. Neither the motor nor the sensory deficit occurrence between the groups was significant (*P*=0.29, *P*=0.92). The only (3.7%) new motor deficit in the MI-TLIF group was a mild quadriceps femoris muscle weakness (4-/5) which improved to 5/5. One sensory deficit (3.7%) occurred in the MI-TLIF group, which was a minimal hypoesthesia in the L4 and L5 dermatomes, and one (3.22%) occurred in the O-TLIF group. This was similar, however, in the L5 and S1 dermatomes. No severe neurological deficit and no cauda equina syndrome occurred in either group.

### 3.2. Quality of Life and Pain Outcomes

Excluding those patients who needed additional surgery due to an adjacent segment disease (three fusion extensions in the O-TLIF group and one in the MI-TLIF group), who suffered from other diseases which would have negatively influenced their quality of life or pain evaluation, and who were unreachable for follow-up, 17 O-TLIF and 21 MI-TLIF cases remained for ODI and VAS comparison (Tables 3 and 4). In the case of the MI-TLIF group, the preoperative ODI was 54 ± 23%, and the postoperative ODI was 18 ± 16%, while in the O-TLIF group the preoperative ODI was 60 ± 18% and the postoperative ODI decreased to 19 ± 18%. While in both MI-TLIF and O-TLIF groups the compared pre- and postoperative ODI scores showed significant improvement (*P* ≤ 0.001 vs. *P* ≤ 0.001), the improvements showed no difference (*P*=0.64) comparing the two techniques. ODI scores improved by 67% in both groups.

VAS low back pain improved from 5.9 ± 4 to 2.5 ± 2.6 in MI-TLIF and from 5.4 ± 4 to1.6 ± 1.8 in O-TLIF group. In both groups, the improvement was significant (*P* ≤ 0.001 vs. *P* ≤ 0.001) as it was in the case of the ODI and the improvements also did not show any difference (*P*=0.47).

VAS leg pain comparison showed also significant (*P* ≤ 0.001 vs. *P* ≤ 0.001) improvement in both groups. VAS leg pain score 7.4 ± 2.6 improved to 1.6 ± 2.5 in the MI-TLIF group and decreased from 5.7 ± 3.4 to 1.6 ± 2.5 in the O-TLIF group. Also the comparison of VAS leg pain scores of the two groups did show no difference (*P*=0.21).

The VAS low back pain scores improved by 70% in the O-TLIF group and by 58% in the MI-TLIF group, while the VAS leg pain scores decreased by 72% vs. 78%.

### 3.3. Learning Curve

Comparing MI-TLIF cases year by year, 03/01/2013–03/31/2018, surgical time duration was the only significant improvement. Descriptive statistics are presented in Table 5.

After the initial surgeries performed in 2013, a few procedures in 2014 had longer operative time, although the difference was insignificant (200 ± 57 to 249.00 ± 1.4 minutes, *P*=0.33). Later on, the length of surgery shortened in 2015 (to 157.4 ± 26.8 minutes) by 43 minutes compared to 2013, although the change was not significant (*P*=0.25). When comparing the mean values of surgical duration times year by year, those mean values had only an insignificant improvement from 2015 through 2018 (157.4 ± 26.8 vs. 136.8 ± 11.8 minutes).

However, significant shortening was noted comparing 2017 and 2018 to 2013 (*P*=0.04, *P*=0.04) and 2014 (*P* ≤ 0.001, *P* ≤ 0.001). The mean value of the operative time dropped by 63 minutes from 2013 until 2018, resulting in approximately 2-hour and 15-minute-long procedures.

Comparing the length of the procedures performed in 2014 to the others, it must be kept in mind that these two procedures still occurred at the beginning of the learning curve and might have been more complex than the others.

No significant difference was revealed regarding the other parameters (LOS *P*=0.56, intraoperative blood loss *P*=0.59, incidence of blood transfusion 0.53, incidental durotomy *P*=0.31, new motor deficit *P*=0.35, and new sensory deficit *P*=0.91) in the overall comparison of all the examined years in the MI-TLIF group.

**Figure 1:**
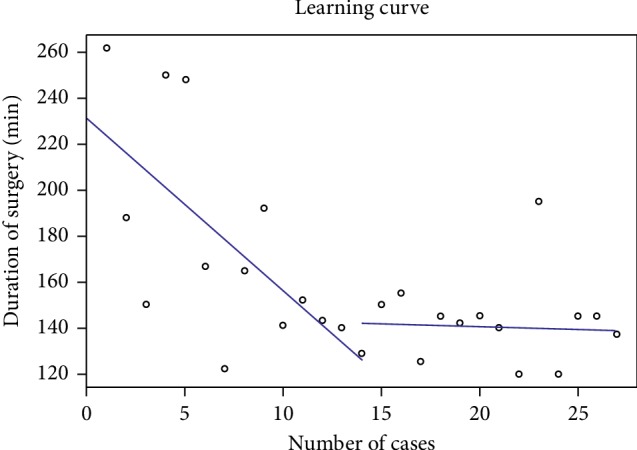
Learning curve is presented on this diagram. Lengths of surgery values are presented in the pre-break point and the post-break point groups of the MI-TLIF cases with linear regression lines. Linear regression lines do not meet because the pre- and post-break group analysis gave two different clusters (due to the statistical modeling) but the results were placed on the same diagram in a second step. After the break point, which is after the 14^th^ case, regression shows decreasing (slightly) tendency.

We examined the change in length of surgery from another point of view. We searched for a break point among the cases from which it became relatively constant, applying Stasinopoulos and Rigby's break point detecting statistical modeling [12]. It requires the analysis to be reduced to an optimization problem. All the possibilities for a break point have to be checked by dividing the cases into two groups. One group contains those cases which are prior to the break point, and the other group contains those which are after the break point. In the two groups, the variance must be calculated, after which the minimalization to the sum must be performed, and that is followed by a linear regression applied to the cases with the lowest values. The break point occurred after the 14th (id42) MI-TLIF case, because at this point *P* is <0.01 (*P*=0.009) comparing the operative time of pre- and post-break point groups and this is the last element of the possible ones after which regression shows decreasing (slightly) tendency (Figure 1).

A comparison of the pre- and post-break point groups regarding other parameters showed that only the LOS changed significantly, resulting in a shorter (1.035 day) hospital stay (*P*=0.09) (Table 6).

## 4. Discussion

The goals of the minimally invasive spine surgery are to reduce the approach-related morbidities achieving similar or better clinical outcomes without increasing the risk of complications and to shorten the recovery time comparing to its open counterpart. Numerous studies compared the outcomes of the MI-TLIF and O-TLIF procedures from different perspectives.

### 4.1. Clinical Outcome

LOS, in its own time frame, should be interpreted with circumspection, because it might be influenced by different health-care-system-related factors. Nonetheless, the observation of LOS as an aspect of recovery and an ultra-short-term clinical outcome marker between the two types of procedures might be considered as a practicable outcome measurement tool. The majority of the articles consistently state that it can be shortened by MI-TLIF [13–19] as our study did. MI-TLIF-treated patients spent less than three days in the hospital, while the O-TLIF group patients spent an average of approximately five days in the hospital. This might suggest that the immediate recovery is faster for those patients who received MI-TLIF.

According to the majority of the literature, ODI and VAS scores favor the MI-TLIF procedures only for a few months after the surgery; however, these scores do not differ measuring at six months or later postoperatively [4, 15, 18]. In our study, the shortest follow-up was longer than one year. ODI, VAS low back pain, and VAS leg pain scores improved significantly in both O-TLIF and MI-TLIF groups, and the improvements did not differ significantly comparing the two types of techniques which correspond to the cited literature.

### 4.2. Length of Surgery

Some found that the MI-TLIF procedure is longer than the O-TLIF procedure [14–16, 20], while others showed that similar operative time can be achieved with either MI-TLIF or O-TLIF [2, 5, 18]. In our study, the operative duration was similar (*P*=0.88) comparing the MI-TLIF to the O-TLIF cohort regarding the entire surgical procedure, approximately 2 hours 20 minutes on average. From 2013 to 2018, the mean MI-TLIF operative time was shortened by 63.3 minutes, providing a mean 136.8-minute procedure length, which is similar to the O-TLIF procedure length which the surgeon achieved in 2017 (140 minutes). Our results show that it is possible to achieve the same surgical speed in MI-TLIF as the given surgeon has in O-TLIF procedures.

### 4.3. Intraoperative Blood Loss

Numerous studies presented that blood loss can be reduced, and even the need for blood transfusion can be decreased by using the MI-TLIF technique over the O-TLIF [14, 18]. Our results fell into the blood loss range given in Goldstein's meta-analysis (51–496 mL in the MI and 125–1147 mL in the open group) [13]. The analysis of our cases showed that blood loss in the O-TLIF group was twice as much as in the MI-TLIF cohort. One patient from MI and two from the O-TLIF group needed blood transfusions (3.7 vs. 6.45%), but there was no significant difference comparing the frequency of blood transfusions (*P*=0.64). However, there was no need for blood transfusions in 2016 and later on in the MI-TLIF group, which might suggest that, with the improvement of surgical skill, the necessity for blood transfusion decreases. We cannot declare that blood transfusion could definitely be avoided using MI-TLIF.

### 4.4. Adverse Events

Authors have suggested that by using MI-TLIF instead of O-TLIF the risk of SSI can be decreased [16, 21]. SSI literature rates show ranges from 0% [16] to 2.45% [21]. The probable reasons behind SSI rate improvement are small surgical corridors, less blood loss, dead space reduction created by less muscle detachment, less muscle retraction resulting in less muscle ischemia, and shorter hospital stays minimizing the exposure to hospital-acquired pathogenic organisms. In our study, two SSIs occurred in the O-TILIF group (6.45%), while there were none (0%) in the MI-TLIF group. Although there was a difference between the two groups favoring MI-TLIF technique, it was not significant (*P*=0.17). Our results suggest that by performing TLIF in a single level in MI style the SSI rate might be reduced, but significant rate decrease is not guaranteed.

According to the literature, the rate of dural tear in O-TLIF procedures ranges from 3 to 17% [22]. A comparison of incidental durotomies also did not show significant differences despite the fact that they occurred twice as often in the O-TLIF (12.9%) than in the MI-TLIF groups (7.4%). Some authors found less dural tear in the MI-TLIF group than in the O-TLIF group, which is consistent with our finding [21, 22]. A possible explanation for fewer incidental durotomies in the MI-TLIF procedures is the utilization of a microscope, which provides the best illumination and magnification for the surgical site. Tormenti, examining 531 cases, found similar durotomy rates as we did in the O-TLIF procedures (14.3%). Interestingly, its rate was not significantly lower in his MI-TLIF group (13.33%) [23].

In our study, there was no statistical difference between the O-TLIF and MI-TLIF groups regarding new motor, sensory deficit, and cauda equina syndrome. There are authors who reported fewer [15, 24] or even higher [19–21, 25] new neurological deficit rates in the MI-TLIF procedures than we found. The high frequency of it could possibly be related to the learning curve, although we could not support it by our data. Overall, both types of procedures appear to have acceptable complication levels.

### 4.5. Learning Curve

As MI spine procedures are becoming more and more popular, the learning curve of the MI-TLIF technique requires attention due to its reported high initial complication rate [19, 21, 25]. An MI-TLIF procedure is a challenging type of surgery, due to a narrow work channel and limited access to the surgical field requiring a profound three-dimensional anatomical knowledge along with excellent fluoroscopic picture assessment, in addition to fine motor dexterity. A learning curve has many aspects and it cannot be characterized only by the shortening of the operative time. In our study, only operative time and LOS showed significant reduction after the surgeon performed more and more MI-TLIF procedures. Lee found that 44 MI-TLIF procedures needed to be performed for a surgeon to achieve technical proficiency [5]. However, according to Phan et al. [17] and Neal and Rosner [6]only 30 and 15 procedures, respectively, were necessary to gain the necessary surgical skills.

We found that the operative time became more or less constant after the 14th (id42) procedure. After that the procedure was performed in approximately 2 hours 25 minutes compared to the first year's time (3 hours 20 minutes). LOS was shortened significantly, by one day, in the post-break point group, meaning that the average hospital stay was reduced to 2 days. In our case, the surgeon, whose learning curve and results are presented in this study, started the MI-TLIF technique and improved his skill on his own. Any learning curve, including the presented one, is highly influenced by the given surgeon's previously and parallel gathered experiences performing other types of spinal procedures, and most certainly depends on the surgeon's individual skills. It must be emphasized that learning curves are highly individual, and the preformation of 14 MI-TLIF procedures to acquire an adequate skill in this technique should not be considered as a standard number.

### 4.6. Limitations

The first limitation of our study is that it is a retrospective, nonrandomized, observational, comparison study, which carries inherent bias. Randomization, which would be a crucial part of a well-founded trial, cannot be achieved since the choice between open or minimally invasive procedure is not based only on the surgeon's decision but on the informed, shared decision making between the patient and the surgeon. This is what is the norm and is widely expected and accepted in the modern era. Second, our study contains the results of a single surgeon of a single centre, and therefore, the generalization of these results is questionable. Additionally, a learning curve is influenced by the given surgeon's other surgical experiences. It would be ideal to observe a surgeon's improvement in only one type of surgical technique, but this is not a realistic expectation. Third, only single-level TLIF procedures were involved into this study, while multilevel procedures might have given different results. Finally, the low number of cases in both groups may affect the results of the present study.

## 5. Conclusions

With an acceptable intraoperative complication rate, similar operative time and postoperative quality of life improvement to O-TLIF procedure can be achieved with a single-level MI-TLIF procedure, while LOS and intraoperative blood loss can be reduced. MI-TLIF can be an alternative option for O-TLIF based on the compared parameters. MI-TLIF learning curve can be steep but can be defined by many factors.

## Figures and Tables

**Table 1:** O-TLIF and MI-TLIF comparison.

	MI-TLIF (*n* = 27)	O-TLIF (*n* = 31)	*P* value

Gender ratio M/F	6/21	14/17	0.07

	Mean ± SD	Mean ± SD	

Age (years old)	56 ± 15	56.4 ± 15.1	0.93
LOS (day)	2.7 ± 1.1	5 ± 2.7	≤0.001
Length of surgery (minute)	161.2 ± 33.7	160 ± 33.6	0.88
Intraoperative blood loss (mL)	288.9 ± 339.8	682.3 ± 465.4	≤0.001

	*n*	*n*	*P* value

Incidence of blood transfusion	1 (3.7%)	2 (6.45%)	0.64

**Table 2:** Adverse events comparison between O-TLIF and MI-TLIF.

	MI-TLIF	O-TLIF	*P* value
	*n*	*n*	
SSI	0	2 (6.45%)	0.17
Incidental durotomy	2 (7.4%)	4 (12.9%)	0.5
New motor deficit	1 (3.7%)	0	0.29
New sensory deficit	1 (3.7%)	1 (3.22%)	0.92
New cauda equina syndrome	0	0	—

**Table 3:** Pre- and postoperative ODI and VAS scores comparison in the O-TLIF and MI-TLIF groups.

	MI-TLIF preoperative	MI-TLIF postoperative	*P* value	O-TLIF preoperative	O-TLIF postoperative	*P* value
	Mean ± SD	Mean ± SD		Mean ± SD	Mean ± SD	
ODI	54 ± 23%	18 ± 16%	*P* ≤ 0.001	60 ± 18%	19 ± 18%	*P* ≤ 0.001
VAS low back pain	5.9 ± 4	2.5 ± 2.6	*P* ≤ 0.001	5.4 ± 4	1.6 ± 1.8	*P* ≤ 0.001
VAS leg pain	7.4 ± 2.6	1.6 ± 2.5	*P* ≤ 0.001	5.7 ± 3.4	1.6 ± 2.5	*P* ≤ 0.001

**Table 4:** Comparison of the differences of the pre- and the postoperative ODI and VAS scores of the O-TLIF and MI-TLIF groups.

	MI-TLIF vs. O-TLIF
	*P* value
ODI pre- & postoperative differences	0.64
VAS low back pain pre- & postoperative differences	0.47
VAS leg pain pre- & postoperative differences	0.21

**Table 5:** Mean and SD values of the examined parameters of the MI-TLIF cases, year by year.

	MI-TLIF						
	2013	2014	2015	2016	2017	2018	Total
	*n* = 3	*n* = 2	*n* = 5	*n* = 3	*n* = 10	*n* = 4	
	mean ± SD	mean ± SD	mean ± SD	mean ± SD	mean ± SD	mean ± SD	mean ± SD
Length of stay postoperatively (day)	3.4 ± 1.4	2.9 ± 0.2	3.3 ± 1	2.5 ± 1.1	2.3 ± 1.1	2.4 ± 1	2.7 ± 1.1
Duration of surgery (minute)	200 ± 57	249 ± 1.4	157.4 ± 26.8	145 ± 6.3	144.6 ± 20.9	136.8 ± 11.8	159.7 ± 39.1
Intraoperative blood loss (mL)	166.7 ± 115.5	400 ± 424.3	530 ± 724.2	183.3 ± 76.4	210 ± 99.4	300 ± 216	288.9 ± 339.8
Incidence of blood transfusion	0	0	0.2 ± 0.4	0	0	0	0.04 ± 0.2
SSI	0	0	0	0	0	0	0
Incidental durotomy	0	0	0	0.3 ± 0.6	0	0.3 ± 0.5	0.74 ± 0.3
New motor deficit	0	0	0	0	0	0.3 ± 0.5	0.04 ± 0.2
New sensory deficit	0	0	0	0	0.1 ± 0.3	0	0.04 ± 0.2
New cauda equina syndrome	0	0	0	0	0	0	0

**Table 6:** Comparison of pre-break point and post-break point groups in the MI-TLIF cohort.

	MI-TLIF		
	Pre-break point group (*n* = 14)	Post-break point group (*n* = 13)	*P* value
	Mean ± SD	Mean ± SD	
LOS (day)	3.2 ± 1.1	2.1 ± 0.8	0.09
Length of surgery (minute)	174.9 ± 46.9	143.4 ± 19	0.33
Intraoperative blood loss (mL)	328.6 ± 457.7	246.2 ± 139.1	0.53
Incidence of blood transfusion	0.1 ± 2.7	0	0.34
SSI	0	0	0
Incidental durotomy	0.1 ± 2.7	0.8 ± 2.8	0.96
New motor deficit	0	0.8 ± 2.8	0.31
New sensory deficit	0	0.8 ± 2.8	0.31
New cauda equina syndrome	0	0	0

## Data Availability

The dataset used and/or analyzed during the current study is included in the Supplementary Materials.

## Abbreviations

O-TLIF: Open transforaminal lumbar interbody fusion
MI-TLIF: Minimally invasive transforaminal lumbar interbody fusion
LOS: Length of stay
MI: Minimally invasive
SSI: Surgical site infection
Tukey's HSD: Tukey's honest significant difference.

## Supplementary Materials

Supplementary Materials In the first sheet, the first column (A) indicates the years when the surgeries were performed. The second column (B) shows the ID number of the given patient who underwent MI-TLIF or O-TLIF procedure. The third column (C) shows the age and the fourth column (D) shows the gender (sex) of the given patient. 1 means male, and 2 means female. In the fifth column (E), the procedures were divided into MI-TLIF versus open TLIF group. 1 means O-TLIF, while 2 means MI-TLIF. The sixth (F), seventh (G), and eighth (H) columns contain the values of the specific surgical parameters which are named in the first row. The unit is defined next to the name of the given parameter. The ninth (I), tenth (J), eleventh (K), twelfth (L), thirteenth (M), and fourteenth (N) columns represent the incidence of each examined parameter. If the examined parameter occurred (i.e., surgical site infection (SSI)) at the given case, then it was marked by ‘yes', and if it did not occur, then it was marked by ‘no'. In the second sheet, there are 3 diagrams which present the comparison of the measured values (LOS, duration of surgery, and intraoperative blood loss) of the MI-TLIF and O-TLIF procedures. In the third sheet, there are 3 diagrams, which show how the mean values of the different parameters (LOS, duration of surgery, and intraoperative blood loss) changed year by year in the case of the MI-TLIF technique. In the fourth sheet, there exist all the data which served for the ODI and VAS comparisons. In the fifth sheet, there exist the data charts of the MI-TLIF and O-TLIF cases separately.
